# Comparison between CHA_2_DS_2_-VASc and the new R_2_CHADS_2_ and ATRIA scores at predicting thromboembolic event in non-anticoagulated and anticoagulated patients with non-valvular atrial fibrillation

**DOI:** 10.1186/s12872-015-0149-3

**Published:** 2015-11-19

**Authors:** Rami Riziq-Yousef Abumuaileq, Emad Abu-Assi, Andrea López-López, Sergio Raposeiras-Roubin, Moisés Rodríguez-Mañero, Luis Martínez-Sande, Javier García-Seara, Xesús Alberte Fernandez-López, Carlos Peña-Gil, Jose Ramón González-Juanatey

**Affiliations:** Cardiology Department, University Clinical Hospital, A choupana s/n, 15706, Santiago de Compostela, Spain

**Keywords:** Atrial fibrillation, Anticoagulant, Thromboembolism

## Abstract

**Background:**

Accurate risk stratification is considered the first and most important step in the management of patients with non-valvular atrial fibrillation (NVAF). We compared the performance of the widely used CHA_2_DS_2_-VASc and the recently developed R_2_CHADS_2_ and ATRIA scores, for predicting thromboembolic (TE) event in either non-anticoagulated or anticoagulated patients with NVAF.

**Methods:**

The non-anticoagulated cohort was comprised of 154 patients, whereas 911 patients formed the cohort of patients on vitamin-K-antagonist. The scores were computed using the criteria mentioned in their developmental cohorts. Measures of performance for the risk scores were evaluated at predicting TE event.

**Results:**

In the non-anticoagulated cohort, 9 TE events occurred during 11 ± 2.7 months. CHA_2_DS_2_-VASc showed significant association with TE occurrence: hazard ratio (HR) = 1.58 (95 % confidence interval [95 % IC] 1.01–2.46), but R_2_CHADS_2_ and ATRIA did not (HR = 1.23 (95 % CI 0.86–1.77) and 1.20 (95 % CI 0.93–1.56), respectively.

In the anticoagulated cohort, after 10 ± 3 months of follow up, 18 TE events were developed. In that cohort, the three scores showed similar association with TE risk: HR = 1.49 (95 % CI 1.13–1.97), 1.41 (95 % CI 1.13–1.77) and 1.37 (95 % CI 1.12–1.66) for CHA_2_DS_2_-VASc, R_2_CHADS_2_ and ATRIA, respectively.

In both cohorts, no TE event occurred in patients classified in the low risk category according to CHA_2_DS_2_-VASc or R_2_CHADS_2_.

**Conclusions:**

In this study of NVAF patients, CHA_2_DS_2_-VASc has better association with TE events than the new R_2_CHADS_2_ and ATRIA risk scores in the non-anticoagulated cohort. CHA_2_DS_2_-VASc and R_2_CHADS_2_ can identify patients at truly low risk regardless of the anticoagulation status.

## Background

Atrial fibrillation (AF) increases the risk of embolic stroke by 5-fold [1]. Effective prevention of thromboembolic events (TE) with oral anticoagulants is the cornerstone of AF management and appropriate TE risk stratification is a critical step in the decision making process regarding this vital issue [2].

The current clinical practice guidelines [3–5] recommend the use of CHADS_2_ [6] and CHA_2_DS_2_-VASc [7] risk scores in the effective TE prevention strategy. CHADS_2_ [6] score was validated and conceived in the year of 2001 with the aim of identifying patients at high risk of TE events. However, patients at low risk according to CHADS_2_ score continued to have significant annual stroke rate [8, 9], this enhanced the motivation to investigate the significance of other risk factors not included in the CHADS_2_ score and, in turn, has led to a clinical shift in the paradigm with a new aim to identify “truly low risk” patients using CHA_2_DS_2_-VASc score [7]. Anyhow, in several studies, CHADS_2_ and CHA_2_DS_2_-VASc showed just a moderate discrimination ability to predict TE complications [10, 11], and at least one recently published large cohort study demonstrated an annual ischemic stroke rate of 1.06 % in the group of patients classified in “the true low risk category” according to CHA_2_DS_2_-VASc [12]. All this could lead to a number of questions and potential avenues for further research.

Recently, and with the aim to improve the ability to predict TE event, two new TE risk scores (i.e. R_2_CHADS_2_ [13] and ATRIA [14]) have been shown, in their own derivation cohorts, better performance than CHADS_2_ and CHA_2_DS_2_-VASc. Really, the two recently proposed risk scores contain new risk factors in their schemes which were not included in the most recommended CHA_2_DS_2_-VASc score. This fact could qualify them to strongly capture the risk of suffering a future TE event, but little information is available in this regard in independent dataset of patients. A recent expert review has announced the need for further validation of the R_2_CHADS_2_ in a real world cohort with full spectrum of estimated glomerular filtration rate (eGFR) [15].

We aimed to evaluate the ability of CHA_2_DS_2_-VASc, R_2_CHADS_2_ and ATRIA scores at predicting TE events in contemporary two different real world cohorts of non-anticoagulated and anticoagulated patients with non-valvular atrial fibrillation (NVAF) which have full spectrum of eGFR.

## Methods

This retrospective study is composed of two separate and different cohorts: for the first cohort, we screened all the consultations which were registered in the emergency department of our tertiary hospital between January 2008 and June 2010, by this we were able to identify all consecutive patients ≥18 years of age with AF documented by electrocardiographic records (*n* = 1873). After excluding patients with prosthetic valve (*n* = 473), rheumatic heart disease (*n* = 46) and/or patients with active cancer (*n* = 61), there were 1293 patients with NVAF. We also excluded patients on anticoagulation (*n* = 1135) and those patients lost to follow up (*n* = 4). Thus, the non-anticoagulated cohort consisted of 154 consecutive patients with NVAF.

The second cohort of the present study was constituted by 911 patients with NVAF on vitamin K antagonists (VKAs), as was previously described [16].

For both cohorts, a detailed medical history was recorded for each patient, and the basal clinical characteristics at study entry together with information on follow up were carefully gathered by cardiologists.

The study was approved by the Clinical Research Ethics Committee of the University Clinical Hospital of Santiago de Compostela. The study protocol conforms to the ethical guidelines of the 1975 Declaration of Helsinki as reflected in a priori approval by the institution’s human research committee.

### TE risk calculation

CHA_2_DS_2_-VASc, R_2_CHADS_2_ and ATRIA scores for predicting TE complications were calculated in each patient from the original corresponding prognostic variables scores used in their derivation cohorts. CHA_2_DS_2_-VASc was calculated by adding 2 points for age ≥ 75 years; 2 points for prior stroke or transient ischemic attack (TIA); and 1 point for each of the following factors: congestive heart failure\left ventricular ejection fraction ≤40 %, hypertension, diabetes mellitus, vascular disease, age 65–74 and female sex, with a maximum score of 9 points.

R_2_CHADS_2_ was calculated by adding 2 points for renal dysfunction (i.e. estimated glomerular filtration rate [eGFR] <60 ml/min/1.73 m^2^); 2 points for prior stroke or TIA; and one point for each of the following factors: congestive heart failure, hypertension, age ≥ 75 and diabetes mellitus with a maximum score of 8 points.

For CHA_2_DS-VA_2_Sc and R_2_CHADS_2_, patients with 0 point were defined as being in the low risk category and patients with 1 point were at intermediated risk, while patients with ≥2 points were in the high risk stratum.

The ATRIA TE risk score was calculated by adding 1 point for each of the following factors: female sex, diabetes mellitus, congestive heart failure, hypertension, proteinuria and renal dysfunction (i.e. eGFR < 45 ml/min/1.73 m^2^ or end-stage renal disease) and by adding 0–9 points depending on the specific score weighting of patients age according to the presence or absence of prior ischemic stroke [14]. We did not have data about proteinuria, so the maximum score of the ATRIA risk score will be 14 points. Patients with ≤ 5 points were defined as low risk category and patients with 6 points were at intermediated risk, while patients with ≥7 points were in the high risk stratum.

eGFR was estimated at study entry for every patient in both cohorts using the 4 variable Modification of Diet in Renal Disease (MDRD-4) [17].

### End point definition

The primary endpoint for the present study was the development of TE event during follow-up. A TE complication was defined as the occurrence of ischemic stroke, TIA or peripheral embolism (including fatal TE events). Diagnosis of stroke or transient ischemic attack required an acute neurological deficit lasting for more or less than 24 h, respectively, which could not be explained by other causes and with at least 1 image test (computed tomography or magnetic resonance) compatible with the diagnosis, as well as confirmation from a neurologist. A diagnosis of peripheral embolism was defined as non-central nervous system embolism leading to an abrupt vascular insufficiency associated with clinical or radiographic evidence of arterial occlusion in absence of another mechanism such as atherosclerosis, instrumentation or trauma.

For both cohorts, patients were followed up for 1-year after the enrolment or until TE event or death occurred. Data on TE event were gathered from the cardiology clinic visits and records, and through hospital files as well as through primary care centers reports. Data regarding death during the follow up period was also recorded.

### Statistical analysis

Qualitative data were expressed as frequencies and percentages while quantitative data were summarized as mean and standard deviation. Each risk score was entered into separate Cox regression models to test their association with TE complication. Thereafter, we calculated the c-statistic as a measure of the predictive ability of the scores and tested the hypothesis that these schemes performed significantly better than chance (indicated by a c-statistic 0.50). We calculated and reported the p-values and hazard ratios (HR) with their 95 % confidence interval (95 % CI). P-value < 0.05 was considered statistically significant. The data was performed using the SPSS v.18 software.

## Results

### Baseline characteristics

Our study enrolled a total of 1065 patients, distributed in two different cohorts. The non-anticoagulated cohort had 154 patients with NVAF and the anticoagulated cohort consisted of 911 patients with NVAF on VKAs.

Table 1 summarizes the baseline characteristics of the patients in each cohort. For the non-anticoagulated cohort the mean age was 74 ± 12 and 52.6 % were females. For the anticoagulated cohort, the mean age was 73 ± 11 and 33.6 % were females.

**Table 1:** Baseline characteristics of patients with nonvalvular atrial fibrillation in the non-anticoagulated and the anticoagulated cohorts

	Non-anticoagulated cohort	Anticoagulated cohort
	*N* = 154	*N* = 911
Age (years)	74 ± 12	73 ± 11
Age ≥65 years, %	127 (82.5)	707 (77.6)
Age ≥75 years, %	75 (40.5)	445 (48.8)
Female sex, %	81 (52.6)	306 (33.6)
Systolic blood pressure at study entry (mmHg)	129 ± 15	139 ± 28
Current smoking, %	53 (30.4)	77 (8.5)
Hypertension, %	110 (71.4)	678 (74.4)
Diabetes mellitus, %	33 (21.4)	220 (24.1)
Peripheral arterial disease, %	20 (12.9)	92 (10.1)
Heart failure, %	10 (6.5)	343 (35.5)
History of stroke or TIA, %	9 (5.8)	103 (11.3)
Coronary artery disease, %	23 (14.9)	127 (13.9)
COPD, %	31 (20.1)	183 (20.1)
Hyperthyroidism, %	2 (1.3)	14 (1.5)
Anemia, %	27 (17.4)	178 (19.5)
Alcohol consumption ≥ 40 gr/daily, %	9 (5.8)	81 (8.9)
Antiplatelets, %	150 (97.4)	23 (2.5)
PINRR (%)	--	58 ± 18
eGFR < 60 ml/min/1.73 m^2^, %	44 (28.6)	311 (34.1)
CHA_2_DS_2_-VASc		
0 point, %	5 (3.2)	62 (6.8)
1 point, %	18 (11.7)	77 (8.4)
≥ 2 points, %	131 (85.1)	772 (84.7)
R_2_CHADS_2_		
0 points, %	22 (14.3)	98 (10.8)
1 points, %	43 (27.9)	142 (15.6)
≥ 2 points, %	89 (57.8)	671 (73.7)
ATRIA		
≤ 5 points, %	79 (51.3)	389 (42.7)
6 points, %	14 (9.1)	115 (12.6)
≥ 7 points, %	61 (39.6)	407 (44.7)

*ATRIA* the anticoagulation and risk factors in atrial fibrillation score, *CHA2DS2-VASc* congestive heart failure, hypertension, age ≥75, diabetes mellitus, prior stroke or transient ischemic attack, vascular disease, age 65–74, female, *COPD* chronic obstructive pulmonary disease, *eGFR* estimated glomerular filtration rate, *PINNR* proportion of international normalized ratio within therapeutic range, *R*_*2*_ *CHADS*_*2*_ renal dysfunction, congestive heart failure, hypertension, age ≥75, diabetes mellitus, prior stroke or transient ischemic attack, *TIA* transient ischemic attack

### Outcomes during follow up

During follow up (11 ± 2.7 months) of the non-anticoagulated cohort, 8 (5.2 %) patients died and 9 (5.8 %) patients developed TE events, 8 of them were ischemic strokes and one event was a peripheral embolic event. For the anticoagulated cohort, 60 (6.6 %) patients died and 18 (2 %) patients developed TE events during the follow up (10 ± 3 months): 13 events were ischemic strokes, 2 events were TIAs and 3 were peripheral embolic events.

### Risk scores performance

#### Risk scores performance in the non-anticoagulated cohort

CHA_2_DS_2_-VASc score classified 85.1 % of patients in the high risk category, while R_2_CHADS_2_ classified 57.8 % as high risk patients; ATRIA classified just 39.6 % of patients in the high risk category (Table 1).

The distribution of the TE events rates in the different risk categories of the three risk scores, demonstrated the absence of occurrence of TE event in the subgroups of patients classified as low risk (i.e. patients with 0 point) according to CHA_2_DS_2_-VASc and R_2_CHADS_2_. However, TE events occurred in 6.3 % of patients classified as at low risk according to ATRIA (Table 2).

**Table 2:** Distribution of thromboembolic events according to the different risk category of each risk score

	Non-anticoagulated cohort	Anticoagulated cohort
	*N* = 9	*N* = 18
CHA_2_DS_2_-VASc		
0 point, %	0 (0)	0 (0)
1 point, %	0 (0)	1 (1.3)
≥ 2 points, %	9 (6.9)	17 (2.2)
R_2_CHADS_2_		
0 point, %	0 (0)	0 (0)
1 point, %	1 (2.3)	0 (0)
≥ 2 points, %	8 (9)	18 (2.7)
ATRIA		
≤ 5 points, %	5 (6.3)	2 (0.5)
6 points, %	0 (0)	1 (0.9)
≥ 7 points, %	4 (6.6)	15 (3.7)

*ATRIA* the anticoagulation and risk factors in atrial fibrillation score, *CHA2DS2-VASc* congestive heart failure, hypertension, age ≥75, diabetes mellitus, prior stroke or transient ischemic attack, vascular disease, age 65–74, female, *R*_*2*_ *CHADS*_*2*_ renal dysfunction, congestive heart failure, hypertension, age ≥75, diabetes mellitus, prior stroke or transient ischemic attack

CHA_2_DS_2_-VASc was the only score to show significant association with TE events: HR = 1.58 (95 % CI; 1.01–2.46). R_2_CHADS_2_ and ATRIA did not show significant association with TE event: HR = 1.23 (95 % CI; 0.86–1.77) and 1.20 (95 % CI; 0.93–1.56) for both scores, respectively (Table 3).

**Table 3:** Association between each risk score as continuous variables and thromboembolic event in both cohorts

	Non anticoagulated cohort	Anticoagulated cohort
	HR (95 % CI)	HR (95 % CI)
CHA_2_DS_2_-VASc	1.58 (1.01–2.46)	1.49 (1.13–1.97)
	*p* = 0.044	*p* = 0.005
R_2_CHADS_2_	1.23 (0.86–1.77)	1.41 (1.13–1.77)
	*p* = 0.25	*p* = 0.03
ATRIA	1.20 (0.93–1.56)	1.37 (1.12–1.66)
	*p* = 0.17	*p* = 0.002

*ATRIA* the anticoagulation and risk factors in atrial fibrillation score, *CHA2DS2-VASc* congestive heart failure, hypertension, age ≥75, diabetes mellitus, prior stroke or transient ischemic attack, vascular disease, age 65–74, female, *CI* confidence interval, *HR* hazard ratio, *NVAF* nonvalvular atrial fibrillation, *p*; *p* value, *R*_*2*_ *CHADS*_*2*_ renal dysfunction, congestive heart failure, hypertension, age ≥75, diabetes mellitus, prior stroke or transient ischemic attack

The discriminative capacity of the three risk scores at predicting TE event are shown in Table 4. CHA_2_DS_2_-VASc and R_2_CHADS_2_ had moderate discriminative capacity with c-statistics of 0.69 (95 % CI; 0.53–0.85) and 0.65 (95 % CI; 0.53–0.78), respectively. The ATRIA score showed a weaker discriminative ability at predicting TE events: c- statistics = 0.64 (95 % CI; 0.49–0.80).

**Table 4:** Discriminatory capacity of risk scores as continuous variables at predicting thromboembolic event in both cohorts

	Non anticoagulated cohort	Anticoagulated cohort
	c-statistics (95 % CI)	c-statistics (95 % CI)
CHA_2_DS_2_-VASc	0.69 (0.53–0.85)	0.72 (0.63–0.82)
R_2_CHADS_2_	0.65 (0.53–0.78)	0.70 (0.61–0.79)
ATRIA	0.64 (0.49–0.80)	0.72 (0.62–0.83)

*ATRIA* the anticoagulation and risk factors in atrial fibrillation score, *CHA2DS2-VASc* congestive heart failure, hypertension, age ≥75, diabetes mellitus, prior stroke or transient ischemic attack, vascular disease, age 65–74, female, *CI* confidence interval, *HR* hazard ratio, *R*_*2*_ *CHADS*_*2*_ renal dysfunction, congestive heart failure, hypertension, age ≥75, diabetes mellitus, prior stroke or transient ischemic attack

#### Risk scores performance in the anticoagulated cohort

CHA_2_DS_2_-VASc score classified 84.7 % of patients in the high risk category, while R_2_CHADS_2_ classified 73.7 % and ATRIA classified just 44.7 % of patients in the high risk category (Table 1).

The distribution of the TE events rates in the different risk categories showed the absence of TE event in patients classified in the low risk category according to CHA_2_DS_2_-VASc and R_2_CHADS_2_. However, two TE events occurred among patients belonged to the low risk category by ATRIA (Table 2).

In terms of hazard ratios, as a measure of association between each risk score and TE events, all the studied scores demonstrated similar and significant association with TE events: HR = 1.49 (95 % CI; 1.13–1.97), 1.41 (95 % CI; 1.13–1.77) and 1.37 (95 % CI; 1.12–1.66) for CHA_2_DS_2_-VASc, R_2_CHADS_2_ and ATRIA, respectively (Table 3).

The three risk scores showed good discriminative ability at predicting TE event: c-statistics = 0.72 (95 % CI; 0.63–0.82) for CHA_2_DS_2_-VASc; 0.70 (95 % CI; 0.61–0.79) for R_2_CHADS_2_ and 0.72 (95 % CI; 0.62-0.83) for ATRIA (Table 4).

## Discussion

In this study comparing three contemporary TE risk scores in non-anticoagulated and anticoagulated real world cohorts of patients with NVAF which have full spectrum of eGFR, CHA_2_DS_2_-VASc was the only score to show significant association in terms of hazard ratio at predicting TE events in the non-anticoagulated cohort.

In the anticoagulated cohort of this study, the three TE risk scores had similar and significant association and discrimination at predicting TE event. On note, only the CHA_2_DS_2_-VASc and R_2_CHADS_2_ were accurate at defining patients at truly low risk to develop TE event in both cohorts.

Oral anticoagulants are highly effective in preventing TE event in patients with AF. However, risk of major bleeding is the downside of oral anticoagulants therapy, so accurate risk estimation of TE event is of paramount importance to help decision making process regarding this issue [2]. Up to our knowledge, this is the first study to compare the most recommended CHA_2_DS_2_-VASc and the recently developed and more sophistically derived R_2_CHADS_2_ and ATRIA scores in real world non-anticoagulated and anticoagulated patients with NVAF.

It is clearly recognized that TE risk scores are best tested in a non-anticoagulated cohort from a real world [15]. In this regard, although R_2_CHADS_2_ and ATRIA contained new risk factors believed to have strong association with TE event like renal dysfunction [13, 14, 18]. However, CHA_2_DS_2_-VASc was the best score to have strong association with TE event in the non-anticoagulated cohort of our study, this may be explained by the fact that factors like renal dysfunction may coexist with advancing age, hypertension, diabetes, heart failure and vascular disease which are already individual components comprising the CHA_2_DS_2_-VASc score. Moreover, our results can be explained and supported if we take into account that R_2_CHADS_2_ score [13] was mainly derived and validated from the ROCKET AF trial of anticoagulated patients which excluded patients with creatinine clearance < 30 ml/min and this may limit its predictability in non-anticoagulated AF patients from the real world with full range of eGFR. Furthermore, similar to our findings in which CHA_2_DS_2_-VASc clearly outperformed ATRIA score in non-anticoagulated cohort of patients with AF, were found in a recent nationwide study [12].

The analysis of the anticoagulated cohort of the current study showed that the three TE risk scores have demonstrated similar association and discrimination at predicting thromboembolism. The improvement we have seen in the performance of the R_2_CHADS_2_ and ATRIA in the anticoagulated cohort may be explained by the fact that factors like renal dysfunction ─which is involved in the R_2_CHADS_2_ and ATRIA─ is a strong independent predictor of poor anticoagulation control and hence for more TE complications [16, 19]. Furthermore, these findings, in turn, may reflect that the non-anticoagulated and the anticoagulated cohorts of patients with NVAF are completely different groups of patients and re-emphasized the belief and strong hypothesis that TE risk scores are best tested in a non-anticoagulated cohort.

In our analysis, CHA_2_DS_2_-VASc and R_2_CHADS_2_ were accurate at identifying truly low risk patients in both cohorts. In previous studies, CHA_2_DS_2_-VASc had identified accurately patients at low risk in non-anticoagulated and anticoagulated patients with NVAF [7, 20]. Similar to our results regarding the reasonable ability of R_2_CHADS_2_ at identifying patients at low risk, were found in the external validation of R_2_CHADS_2_ in which the rates of TE event in the low risk patients at 3-years of follow up were as low as 0.36 % and 0.5 % in the non-anticoagulated and anticoagulated subgroups of the ATRIA study cohort, respectively [13].

In the two different cohorts of current study, ATRIA classified about half of patients in the low risk category, and this may limit its ability to correctly classify patients at truly low risk. Similar performance of the ATRIA risk score was found in a recent study enrolled large cohort of patients [12].

Similar to our findings in which CHA_2_DS_2_-VASc classified the greatest number of patients as being at high risk (85.1 %) and (84.7 %) in the non-anticoagulated and anticoagulated cohorts, respectively, were reported previously [12, 20]. This may aid and reflect the accuracy of CHA_2_DS_2_-VASc at classifying a small group of patients who are truly at low risk of TE event.

Finally, our analysis of the anticoagulated cohort showed that those patients in the high risk category according to CHA_2_DS_2_-VASc and the R_2_CHADS_2_ are still at high risk of developing TE event despite anticoagulation. Really, the identification of patients who remain at high risk of TE event despite anticoagulation could be of great importance in daily clinical practice as this high risk group of patients may need specific treatment strategy with close follow up and more efforts to improve the quality of anticoagulation control and to achieve the best management of their risk factors like hypertension, diabetes and heart failure.

Although this is the first study aimed to compare the CHA2DS2-VASc and the new R2CHADS2 and ATRIA scores in real world non-anticoagulated and anticoagulated cohorts of patients with NVAF. However, the relatively small number of patients enrolled in the current study - when compared with several previous studies [12–14] - might limit the validity of our results. This might reflect the need for future studies with large cohorts of patients for further validation of the interesting results obtained from our analysis.

Our overall results when taken together might indicate that the CHA_2_DS_2_-VASc still the best user friendly tool at predicting TE event as well as at identifying patients at truly low risk particularly in the non-anticoagulated patients who are actually need accurate TE risk stratification.

### Limitations

The main limitation of our study is its retrospective design, but it has interesting strong points as it reflects real world practice by enrolment of two contemporary separate and different cohorts of non-anticoagulated and anticoagulated patients with NVAF consulted the emergency department or the outpatient cardiology clinics of a tertiary hospital with the advantage of careful follow-up and data collection by cardiologists. Nevertheless, in this regard prospective studies in the future may be needed for better assessment of the clinical validity of our results.

The sample size of the non-anticoagulated cohort of the current study might be another limitation of our study that could limit the likelihood of detecting small effects or significant relationships from the data. However, the availability of a contemporary large non-anticoagulated cohort of patients with NVAF is challenging and increasingly unlikely. Furthermore, the findings in our study might need to be enhanced by further studies with large real world cohorts of patients with NVAF. In the non-anticoagulated cohort, the vast majority of patients were taking antiplatelet therapy during follow up. However, antiplatelet therapy alone is not a substitute for thromboembolic prevention in AF and could not reduce significantly the TE risk [21], so patients in the non-anticoagulated cohorts continue to have high TE risk during the follow up.

Really, most patients in the non-anticoagulated cohort were at risk of TE event and despite this, the anticoagulation was underused in these patients. This may be mainly due to the effect of advance age, associated comorbidities and/or patient preference on the medical decisions taken by the emergency department doctors and the reluctance to change the medication regime.

## Conclusions

CHA_2_DS_2_-VASc has better association with TE events than R_2_CHADS_2_ or ATRIA in non-anticoagulated patients with NVAF, and represents in this study the more accurate clinical tool for TE risk stratification in these patients. The CHA_2_DS_2_-VASc and the R_2_CHADS_2_ scores may accurately identify patients at truly low risk of developing future TE events regardless of the anticoagulation status.

## Availability of supporting data

The database is available in the department of Cardiology- University Clinical Hospital of Santiago de Compostela and needs authorized access. The original dataset is available on request from the corresponding author at drrami2012@hotmail.com.

## Abbreviations

AF: atrial fibrillation
eGFR: estimated glomerular filtration
HR: hazard ratio
MDRD-4: 4 variable modification of diet in renal disease
NVAF: nonvalvular atrial fibrillation
TE: thromboembolic event
TIA: transient ischemic attack
VKAs: vitamin k antagonists
